# Molecular detection of urogenital *mollicutes* in patients with invasive malignant prostate tumor

**DOI:** 10.1186/s13027-021-00344-9

**Published:** 2021-01-20

**Authors:** Osama Mohammed Saed Abdul-Wahab, Mishari H. Al-Shyarba, Boutheina Ben Abdelmoumen Mardassi, Nessrine Sassi, Majed Saad Shaya Al Fayi, Hassan Otifi, Abdullah Hassan Al Murea, Béhija Mlik, Elhem Yacoub

**Affiliations:** 1 Department of Clinical Laboratory Sciences, Faculty of Applied Medical Sciences, King Khalid University, Abha, Saudi Arabia; 2 Department of Surgery, College of Medicine, King Khalid University, P.O. Box 641, Abha, 61421 Saudi Arabia; 3 Specialized Unit of Mycoplasmas, Laboratory of Molecular Microbiology, Vaccinology, and Biotechnology Development, Institut Pasteur de Tunis, University of Tunis El-Manar, Tunis, Tunisia; 4 Department of Clinical Laboratory Sciences, Faculty of Applied Medical Sciences, King Khalid University, P.O. Box 641, Abha, 61421 Saudi Arabia; 5 Department of Pathology, College of Medicine, King Khalid University, P.O. Box 641, Abha, 61421 Saudi Arabia; 6 Laboratory Department, Aseer Central Hospital, P. O. Box 34, Abha, 51411 Saudi Arabia

**Keywords:** Prostate cancer, Urogenital mollicutes, PCR, Sequencing

## Abstract

**Background:**

The etiology of prostate cancer (PCa) is multiple and complex. Among the causes recently cited are chronic infections engendered by microorganisms that often go unnoticed. A typical illustration of such a case is infection due to mollicutes bacteria. Generally known by their lurking nature, urogenital mollicutes are the most incriminated in PCa. This study was thus carried out in an attempt to establish the presence of these mollicutes by PCR in biopsies of confirmed PCa patients and to evaluate their prevalence.

**Methods:**

A total of 105 Formalin-Fixed Paraffin-Embedded prostate tissues collected from 50 patients suffering from PCa and 55 with benign prostate hyperplasia were subjected to PCR amplification targeting species-specific genes of 5 urogenital mollicutes species, *Mycoplasma genitalium, M. hominis, M. fermentans, Ureaplasma parvum*, and *U. urealyticum*. PCR products were then sequenced to confirm species identification. Results significance was statistically assessed using Chi-square and Odds ratio tests.

**Results:**

PCR amplification showed no positive results for *M. genitalium, M. hominis,* and *M. fermentans* in all tested patients. Strikingly, *Ureaplasma* spp. were detected among 30% (15/50) of PCa patients. Nucleotide sequencing further confirmed the identified ureaplasma species, which were distributed as follows: 7 individuals with only *U. parvum*, 5 with only *U. urealyticum*, and 3 co-infection cases. Association of the two ureaplasma species with PCa cases proved statistically significant (*P* < 0.05), and found to represent a risk factor. Of note, *Ureaplasma* spp. were mostly identified in patients aged 60 and above with prostatic specific antigen (PSA) level > 4 ng/ml and an invasive malignant prostate tumor (Gleason score 8–10).

**Conclusions:**

This study uncovered a significant association of *Ureaplasma* spp. with PCa arguing in favour of their potential involvement in this condition. Yet, this finding, though statistically supported, warrants a thorough investigation at a much larger scale.

**Supplementary Information:**

The online version contains supplementary material available at 10.1186/s13027-021-00344-9.

## Background

Cancer is a major public health issue, considered as one of the leading causes of death worldwide. According to latest records of the International Agency for Research on Cancer, World Health Organization (WHO), the Prostate Cancer (PCa) ranked fourth among the most common cancers worldwide with 1.28 million cases [1]. The main established risk factors for PCa are increasing age, ethnicity, and family history [2], yet compelling evidence suggests a role for environmental factors and infections [3]. In fact, it was found that chronic inflammation associated with infections could tip the balance towards abnormal cellular growth and proliferation. The mechanisms governing the inflammation process that triggers cancer development are beginning to be investigated. In prostate cancer for example, inflammatory reaction was found to be orchestrated by a multiprotein complex known as inflammasomes. Once activated with an infection and/or another stimulus (cell injury, hormonal variation, dietary or environmental factors …), these inflammatory regulators induce a cascade of proinflammatory signaling molecules capable of modulating different cellular activities. This often results in cell damage and enhances abnormal cell proliferation [4]. Thus, inflammation has been considered as an important carcinogenesis promoting condition for some organs, including prostate [5, 6]. Different types of cancer such as those of colon, stomach, liver, and prostate have been linked to persistent inflammation due to chronic infections [7–9]. Additionnaly, according to the American Cancer Society, about 15 to 20% of cancers worldwide are directly related to infectious agents [10]. For oncoviruses for example, the causal relationship with cancer has been well documented since the early 1980s. Since then, viruses are admitted as bona-fide causes of many human cancer types [11, 12]. Apart from viruses, the link between bacterial infections and the development of malignancy was also well explored. It was suggested that several prokaryotes are able to exhibit tumorigenic properties [13, 14]. For instance, many convincing proofs have attributed *Streptococcus bovis* to colon cancer [15], *Salmonella thyphii* to gallbladder cancer [16], *Chlamydia pneumoniae* to lung cancer [17], *Bartonella* species to vascular cancer [18], and *Helicobacter pylori* to gastric cancer [19, 20]. Yet, while the latter association has arguably been established [21], it is still unclear whether these infections are themselves directly involved in tumorogenesis or represent a promoting factor.

Regarding mycoplasmas, the smallest known free-living bacteria, their oncogenic potential and role in cancer development have been investigated as early as the 1960s [22]. Actually, the association between malignancy and human mycoplasmas was initially suggested for the human respiratory pathogen *Mycoplasma pneumoniae,* which was among the first species suspected to be associated with human leukemia [23]. Since then, many other studies suggesting a possible role for mycoplasmas in malignancy have been reported, but no evidence of causation has been brought forward [24]. However, the potential to cause cancer of other mycoplasma species, such as *M. fermentans*, *M. penetrans*, and *M. hyorhinis* has been demonstrated in vitro [25–27]. Additionally, it was also shown that mycoplasma infection is able to transform embryonic cells in mouse by promoting the expression of some oncogenes like H-*ras* and c-*myc*. Prolonged infection with mycoplasma was found to lead to prominent chromosomal alteration (rearrangement, translocation, altered pattern of DNA …), to morphological changes, and to uncontrolled cell growth [28]. For *M. hominis*, *M. genitalium*, and *U. urealyticum*, a potential involvment in PCa has been suggested since these species were detected by PCR in patients suffering from this type of cancer [29–33]. These findings are in line with the ability of these mycoplasma species to establish chronic infections in individuals with prostate cancer as evoked since the mid-twentieth century [34, 35].

A rising incidence of PCa was observed in Asian patients from China, India, and Malaysia [36, 37]. It’s worth mentioning that this type of cancer was ranked second most commonly diagnosed cancer and six leading cause of cancer-related death in men worldwide. Likewise, the situation in the Kingdom of Saudi Arabia (KSA) regarding PCa incidence was considered critical as the statistics were getting worse. Indeed, according to the Saudi Cancer Registry and referring to local literature, an upward trend in PCa incidence, from 2.7% in 1994 to 6.3% in 2008, was reported [38, 39]. Although alarming in the KSA, this serious disease is neither studied enough nor the related data being updated.

Here, we investigated the possible involvment of urogenital mollicutes in PCa in a cohort of Saudi patients from Aseer region. We sought to demonstrate the presence of *M. genitalium*, *M. hominis*, *M. fermentans*, *U. urealyticum*, and *U. parvum* by PCR amplification targeting species-specific genes.

## Methods

### Patients

A total of 105 Saudi patients with inflamed, swelling, and painful prostate gland, referred to Aseer Central Hospital (Abha, southwestern KSA) during the years 2010 to 2017, were enrolled in this work. All patients were presenting suspicious and abnormal digital rectal examination (DRE) findings and/or elevated PSA (Prostate Specific Antigen) level (≥ 4 ng/ml). Patients age ranged from 46 to 104 years. Data about Gleason score and PSA were recorded.

### Prostate specimens preparation

Prostate specimens were obtained from patients either by transrectal ultrasound (TRUS)-guided biopsy or by surgery (transurethral resection of the prostate: TURP). Both procedures were carried out by specilized physicians in the Central Hospital of Aseer Region (Abha town, KSA).

TRUS biopsy was performed using an endfiring 7.5-MHz biplane transrectal probe attached to an ultrasonography device (bk 3000 ultrasound systems, Denmark). No local anesthetic injection was used as most of the patients tolerated the procedure very well. However, all of them received a general pain killer (75 mg intramuscular pethidine (demerol) injection pre-procedural statin). Biopsy was performed half an hour after pethidine injection. To prevent patients from post-interventional complications, three oral doses of Ciprofloxacillin (500 mg) were administrated to them before, during, and after the biopsy procedure.

TURP was undertaken by urological surgeons. The procedure was carried out under general anaesthesia using bipolar devices. Gentamicin antibiotic (160 mg) was administrated intravenously to patients 1 h before the TURP surgical procedure.

### Histopathological examination

All patients specimens were placed in biocassettes and were dehydrated in histokinette with 10% formalin solution for 24 h. Then, they were embedded in paraffin blocks, sectioned with a microtome into slices of 4 μm thick and stained with hematoxylin-eosin (H & E). Differentiation between the benign and malignant prostatic specimens, as well as the aggressiveness classification of the cancerous tissues according to Gleason score [40] were done by experienced Saudi pathologists using light microscopy. Overall, 50 patients were diagnosed with prostate cancer (Cohort 1). Based on anatomopathological examination and Gleason score attribution, tumors were defined as aggessive (poorly differentiated, Gleason score 8–10) in 38 patients and as non-aggessive (moderately differentiated, Gleason score 6–7) in 12 patients. The other 55 patients were diagnosed with Benign Prostate Hyperplasia (BPH) and used as negative controls (Cohort 2).

Formalin-Fixed Paraffin-Embedded (FFPE) prostate tissues were forwarded to the laboratory of mycoplasmas, Institut Pasteur, Tunis, for further manipulations.

### DNA isolation from prostate specimens and from mycoplasma cultures

Total genomic DNA was extracted from sections of FFPE prostate tissue using the QIAamp® DNA FFPE Tissue Kit (Qiagen, Germany) according to the supplier’s instructions. For each biopsy, up to eight sections of 5 to 10 μm thick were cut using a microtome and placed in a sterile 2-ml microcentrifuge tube. Sections of FFPE prostate tissue were subjected to paraffin dissolution using xylene (Honeywell, France), followed by homogenization and enzymatic digestion with proteinase K and lysis buffer. Samples were incubated overnight at 56 °C and lysates were purified using the QIAamp MinElute columns to obtain high quality DNA for PCR.

Using the classical phenol/chloroform DNA extraction method [41], genomic DNA was also extracted from the urogenital mollicutes species, *M. hominis* PG21 (ATCC 23114), *M. genitalium* G37 (ATCC 33530), *M. fermentans* PG18 (ATCC 19989), *U. urealyticum* (ATCC 27618), and *U. parvum* (ATCC 27813), in order to be used as controls in the PCR reactions. These mollicutes species were cultivated on appropriate SP4 broth medium, prepared according to the previously described formulation [42]. Antigen of each species was collected and washed by a series of centrifugations at 16000 rpm at 4 °C for 30 min using PBS buffer.

Quantity and quality of DNA were assessed by Nanodrop absorbance measurement and by electrophoresis on 1% agarose gel stained with CYBR safe. The obtained purified DNAs were stored at − 20°С until needed.

### Standard PCR assays for screening of urogenital mollicutes DNA in the FFPE samples

For each mollicutes species, PCR assays were designed to amlify 16S rRNA gene along with a specific gene fragment; *p120’* gene (coding for DUF885 family protein) for *M. hominis*, *mgpA* gene (coding for Adhesin P1 protein) for *M. genitalium*, the gene conding for the Insertion Sequence like-element (IS*1630I*) for *M. fermentans*, and *ureB* gene (coding for beta subunit of the urease enzyme) for *Ureaplasma* spp. An internal control specific to the human mitochondrial cytochrome c oxidase subunit III gene (named MT-CO3) was also included. Details about these target genes and their respective primer sequences are presented in Table 1. PCRs were performed in a total volume of 50 μl in a thermal cycler (Applied biosystems, USA model 2720). All PCR mixtures contained 10X *Taq* buffer, 1 mM MgCl_2_, 0.2 mM dNTP (Sigma, Germany), 1 pmol of each primer, 1.5 U of HotStarTaq DNA polymerase (Qiagen, Germany), and 1–2 μl (approximately 150 ng) of genomic DNA. All samples were subjected to a first denaturation step at 95°С for 10 min and then to 35 cycles at 94°С for 1 min (denaturation), 55°С for 1 min (annealing), and 72°С for 1 min (elongation). The cycling was ended by an extension step at 72 °C for 10 min. Ten microliters of each amplicon were electrophoresed on a 2% agarose gel stained with CYBR safe and visualized under UV light.

**Table 1:** Characteristics of primers used for detection of mollicutes investigated in this study

Mycoplasma species	Primer designation	Target gene	Gene locus	Product	Amplicon size (bp)	Sequence (5′-3′)	References
*M. hominis*	Mhp120’F Mhp120’R	*p120’*	MHO_RS02125	DUF885 family protein	190	GTTTTAGAACGTAAAATTCTA CATCAGCAGGATTGTTTAAAGCT	This study^a^
	Mh16SF Mh16SR	*rrnA16S*	MHO_RS01540	16S ribosomal RNA	164	GGTAATGGCCCACCAAGACTAT GAAGACCTTCATCGTGCACGCTG	This study^a^
*M. genitalium*	MgPa335F MgPa432R	*mgpA*	MG_RS01075	Adhesin protein	78	GAGAAATACCTTGATGGTCAGCAA GTTAATATCATATAAAGCTCTACCGTTGTTATC	[43]
	Mg16SF MG16SR	*rrnA16S*	MG_RS00775	16S ribosomal RNA	195	TAGCTAATACCGCATAAGAACTTT CTCCCGTAGGAGTATGGGCCGTGT	This study^a^
*M. fermentans*	RW 005 RW 004	IS*1630I*	MBIO_RS03870	IS30-like element	205	GGTTATTCGATTTCTAAATCGCCT GGACTATTGTCTAAACAATTTCCC	[44]
	Mf16SF Mf16SR	*rrnA16S*	MBIO_RS4090	16S ribosomal RNA	186	TTGACGGTACCTTATTAGAAA TCTACACAACTCTAGCCTG	This study^a^
*U. urealyticum*	UUureF UUureR	*ureB*	UUR8_RS02385	Urease subunit beta	100	GATCACATTTCCACTTATTTGAAACA AAACGACGTCCATAAGCAACTTTA	[45]
	Uu16SF Uu16SR	*rrnA16S*	UUR8_RS00640	16S ribosomal RNA	181	TGCCTGGGTAGTACATTCGCAAGAA GACAACCATGCACCACCTGTC	This study^a^
*U. parvum*	UPureF UPureF	*ureB*	UPA3_RS02275	Urease subunit beta	99	GATCACATTTTCACTTGTTTG AAGTG AACGTCGTCCATAAGCAACTTTG	[45]
	Up16SF Up16SF	*rrnA16S*	UPA3_RS00595	16S ribosomal RNA	183	CTTTTATATGGGAAGAAACGCT GCATCTAGATTTAATACC	This study^a^
*Human DNA*	HmitoDNAF HmitoDNAR	*MT-CO3*	YP_003024032.1	Cytochrome c oxidase subunit III	208	GGATAATCCTATTTATTACCTCAGAAG AGACTATGGTGAGCTCAGGTGATTGAT	This study^a^

^a^Primers for each gene were designed based on sequences available in GenBank

### Sequencing and bioinformatics analysis

PCR products were cleaned-up using the ExoSAP protocol. For this purpose, exonuclease I (Biolabs, England) and shrimp alkaline phosphatase (Biolabs, England) enzymes were used to remove leftover primers and remaining dNTPs, respectively. Sequencing was performed using the BigDye Terminator v3.1 cycle sequencing kit (Applied Biosystems, USA), according to the manufacturer’s instructions. Sequencing products were run in Applied Biosystems® 3130 Genetic Analyzer (USA) available at the sequencing facility of Institut Pasteur, Tunis. For each PCR amplicon, both strands were sequenced. The derived nucleotide sequences were aligned with reference sequences downloaded from GenBank using the BioEdit Sequence Alignment Editor, version 7.2.5.

### Statistics

Statistical analyses were performed using Excel tools (Microsoft, USA) to assess the prevalence of mollicutes species among the studied population. The chi-square test was computed to evaluate the potential relationship between the urogenital mollicutes infections and PCa. *P* value < 0.05 was considered statistically significant. Odds ratio with a 95% confidence interval was also calculated to estimate the role of mollicutes as a risk factor in PCa disease. Odds ratio values > 1 indicated that mollicutes infections might be a risk factor for the PCa. All these tests were performed using the Statistical Package for Social Science (SPSS) 20.0 software (IBM Corp. Armonk, NY, USA).

## Results

The mean age of Saudi men included in this study was 76.08 years for those suffering from PCa and 72.18 years for those diagnosed with BPH. Within both groups, patients aged between 70 and 79 years were the most frequent (46 and 40%, respectively). In both groups, the least common patients were those aged under 60, particularly in the PCa category with only 2 cases (4%). Regarding PSA records, levels were > 4 ng/ml in 92% (48/50) of patients with PCa and 70% (38/55) of patients displaying BPH. Interestingly, high PSA levels exceeding 20 ng/ml were recorded for a large number of PCa patients (62%), whereas only 13% amongst BPH cases showed such high levels. After anatomopathology examination, Gleason score attribution was only applied to patients suffering from PCa. Patients with aggressive PCa were found to be nearly three times more frequent than those with non-aggressive PCa (76% versus 24%, respectively). The characteristics of the study population are summarized in Table 2.

**Table 2:** Characteristics of the studied population

	PCa (*n* = 50)		BPH (*n* = 55)	
Age (years)				
< 60	2	(4%)	5	(9%)
60–69	9	(18%)	17	(31%)
70–79	23	(46%)	22	(40%)
> 79	16	(32%)	11	(20%)
PSA level (ng/ml)				
0–4	4	(8%)	17	(31%)
4.1–10	7	(14%)	17	(31%)
10.1–20	8	(16%)	14	(25%)
> 20	31	(62%)	7	(13%)
Gleason score				
6–7	12	(24%)	–	–
8–10	38	(76%)	–	–

*PCa* Prostate Cancer, *BPH* Benign Prostate Hyperplasia, *PSA* Prostate Specific Antigen

**Fig. 1:**
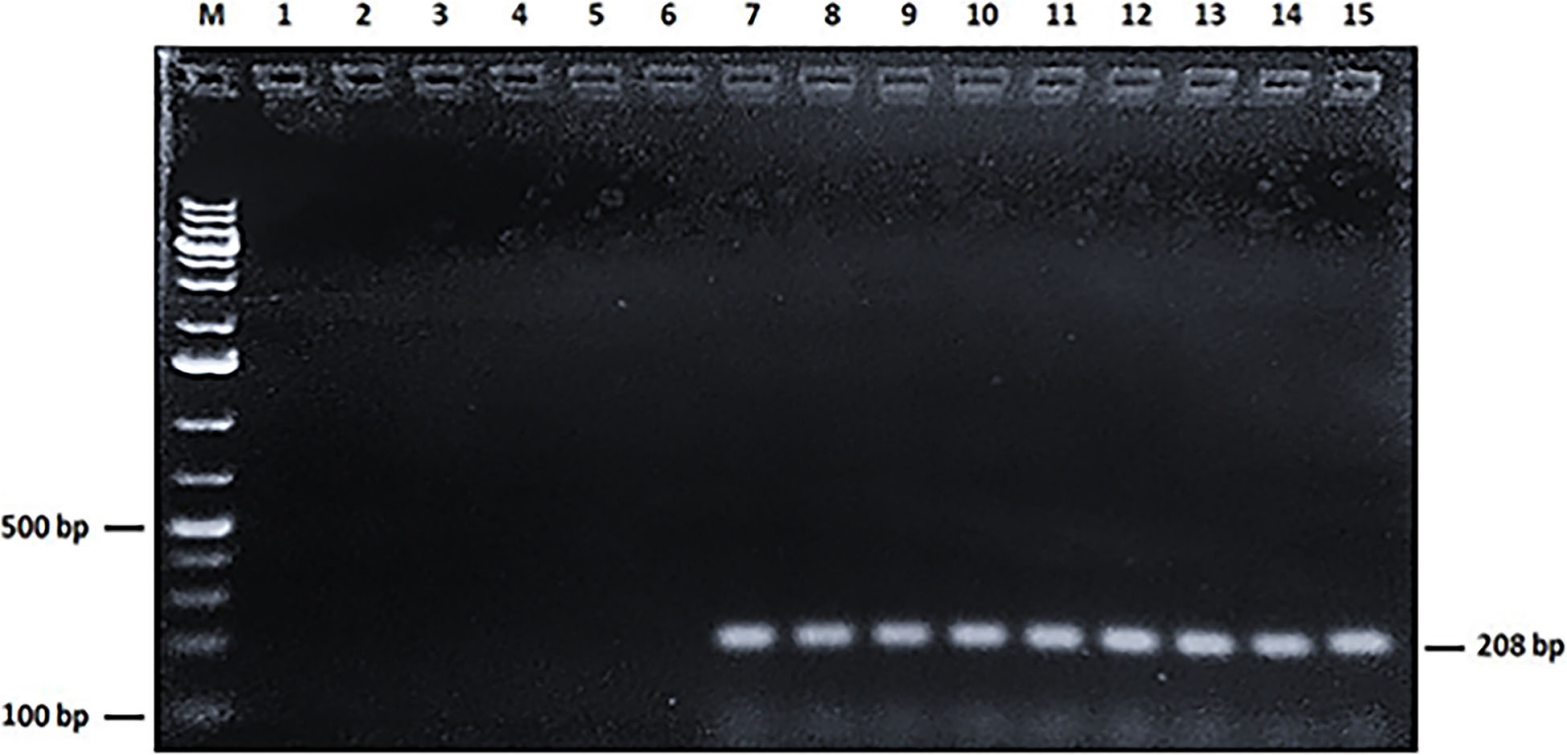
PCR amplification of the 208-bp fragment of human mitochondrial DNA (internal positive control). Lane M: 1Kb + DNA ladder. Lane 1–5: Specificity of the human mitochondrial DNA primers with genomic DNA from *M. genitalium*, *M. hominis*, *M. fermentans*, *U. parvum*, and *U. urealyticum*, respectively. Lane 6: No DNA (negative control). Lane 7: Human DNA (positive control). Lane 8–15: DNA from prostate biopsies

Electrophoresis and Nanodrop quantification have shown that genomic DNA extracted from all mollicutes reference strains as well as DNA collected from all FFPE human samples were of high quality and quantity (DNA concentration ranged from 100 to 1750 ng/μl). This has been confirmed by the efficient amplification of a 208-bp fragment of the human mitochondrial DNA. Indeed, all the 105 samples (100%) produced this targeted fragment at its expected size (Fig. 1).

**Fig. 2:**
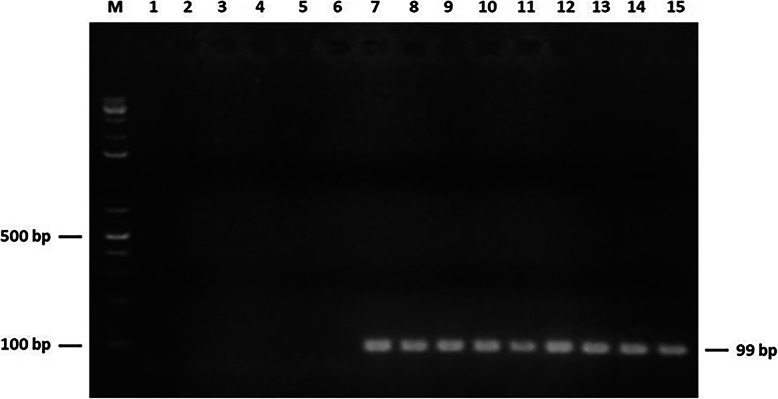
PCR detection of the 99-bp fragment of *Ureaplasma parvum ureB* gene. Lane M: 1 kb + DNA ladder. Lanes 1–5: Specificity of primers of *U. parvum ureB* gene with DNA from human mitochondrion, *M. hominis*, *M. fermentans*, *M. genitalium*, and *U. urealyticum*. Lane 6: No DNA (negative control). Lane 7: DNA from *U. parvum* type strain ATCC 27845 (positive control). Lanes 8–15: DNA from prostate biopsies of patients (PCa cohort)

**Fig. 3:**
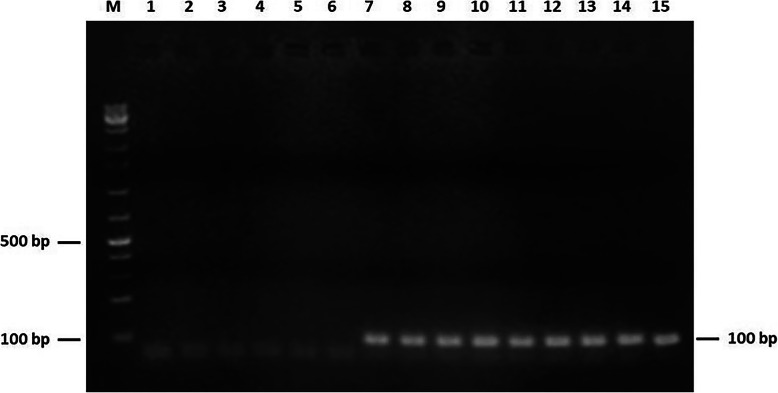
PCR amplification of the 100-bp fragment of *Ureaplasma urealyticum ureB* gene. Lane M: 1 kb + DNA ladder. Lanes 1–5: Specificity of primers of *U. urealyticum ureB* gene with DNA from human mitochondrion, *M. hominis*, *M. fermentans*, *M. genitalium*, and *U. parvum*. Lane 6: No DNA (negative control). Lane 7: DNA from *U. urealyticum* type strain ATCC 27618 (positive control). Lanes 8–15: DNA from prostate biopsies of patients (PCa cohort)

None of the 105 prostate samples was found to contain DNA of mycoplasma species. Indeed, all PCR amplifications targeting 16S rRNA and specific genes of *M. genitalium, M. hominis,* and *M. fermentans* were negative (Data not shown). Interestingly, PCR-based detection of ureaplasma species was positive in 18 cases. The biotyping of *Ureaplasma* spp. revealed *U. parvum* and *U. urealyticum* in ten (20%) and eigth (16%) samples, respectively. The 99-bp and 100-bp PCR products corresponding to the amplification of *U. parvum* and *U. urealyticum* specific urease coding genes are shown in Figs. 2 and 3, respectively. This result was further confirmed by positive amplification of the targeted segments within 16S rRNA gene in *Ureaplasma* spp. (Figures S1 and S2). Both forward and reverse strands of these PCR amplicons were sequenced. Alignement of the derived nucleotide sequences with corresponding *Ureaplasma* spp. reference sequences showed a 100% identity and thus confirmed species identification. Of note, these ureaplasma-positive PCR were only observed in prostate samples of patients diagnosed with PCa. Chi-square test was computed for each ureaplasma species to assess the significance of its potential association with PCa. PCR results relative to these two species in both cohorts group patients were taken into account. Both calculated *P* values proved statistically significant (*P* = 0.0005 for *U. parvum* and *P* = 0.002 for *U. urealyticum*). The Odds Ratio values were found to be infinite, suggesting that infection with each of ureaplasma species could constitute a risk factor in PCa disease. Results of mollicutes detection in archival tissues are summed up in Table 3.

**Table 3:** Prevalence of urogenital *Mollicutes* identified in biopsies of prostate with malignant or benign cancer

Target organism	Overall prevalence				*P* value
	Malignant tumor tissues		Benign tumor tissues		
	***n*** = 50	%	***n*** = 55	%	
*Ureaplasma parvum*	10	20	0	0	0.0005
*Ureaplasma urealyticum*	8	16	0	0	0.002
*Mycoplasma hominis*	0	0	0	0	NA
*Mycoplasma genitalium*	0	0	0	0	NA
*Mycoplasma fermentans*	0	0	0	0	NA

*NA* Not applicable

The 18 *Ureaplasma* spp. positive PCR were detected in 15 patients with PCa. These positive cases were distributed as follows; 7 patients were found to be infected with only *U. parvum*, 5 with only *U. urealyticum*, and 3 were co-infected with both species. Based on their Gleason score, tumors of most of the PCa patients infected with *U. urealyticum* are categorized as aggressive (7 out 8 patients; 87.5%). For those infected with *U. parvum*, the rate of aggressive tumors was also high (7 out of 10 patients; 70%).

**Fig. 4:**
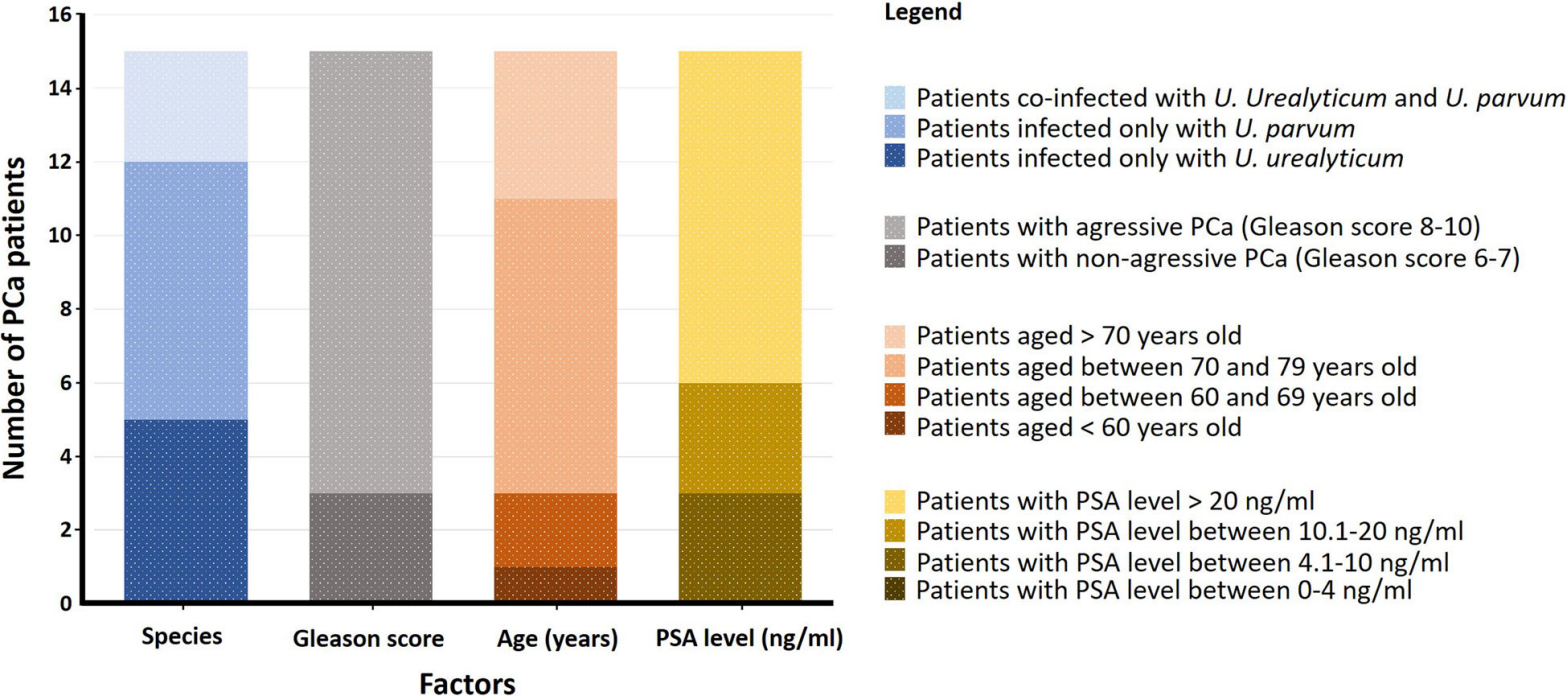
Distribution of the 15 patients diagnosed with PCa and found to be infected with *Ureaplasma* species (Y-axis) on the basis of various factors (X-axis)

Considering both ureaplasma species and taken into account the three cases of co-infection, statistics showed that among the 15 patients in which *Ureaplasma* spp. were detected, 12 were diagnosed with an aggressive PCa (Gleason score between 8 and 10), which represents 80% (12/15). These 15 patients were found to belong to different age intervals. Aside from one PCa patient, all the others (14/15; 93.33%) are aged more than 60 years. Moreover, the majority of these patients (9/15; 60%) showed high PSA levels, exceeding 20 ng/ml. The caracterization based on different parameters (Species, Gleason score, age, and PSA level) of the group of patients diagnosed with PCa and found to harbor *Ureaplasma* spp. DNA is presented in Fig. 4.

## Discussion

Because of its wide-ranging impact on the human health and social life, cancer has prompted scientific research to delve into its causes and risk factors. Millions of new cases and deaths are recorded worlwide every year [46]. Several studies on determinants of cancer’s origins have led to the suspicion that chronic infections are involved in its establishment or progression [47, 48]. In the begining, this assumption was always contreversial given that cancer is usually assumed to have a genetic background. Over the last few decades, the biology and mechanisms governing the cancer-infectious agents relationship are beginning to be unraveled. One of these mechanisms is the infection-related inflammation. For example, the activation of the nuclear factor kappaB (NF-κB) inflammatory pathway, involved in the maintenance of the cell cycle stability, was associated to cancer. This activation could inhibit apoptosis and promote cell transformation. This was reported for the bacterium *Helicobacter pylori* in the case of gastric cancer [49] as well as for many oncoviruses like hepatitis virus [50].

While the role of viruses in carcinogenesis has long been established, the possible involvement of bacteria is beginning to gain more and more acceptance [51–57]. With regard to prostate cancer, it was suggested that sexually transmitted infections could be a risk factor for its developement [58]. Recent advances in techniques of molecular microbiology have allowed the identification of many microorganisms in prostate cancer tissues, such as the sexually transmitted bacteria *Chlamydia trachomatis* and *Trichomonas vaginalis*, the prostatitis-associated *Escherichia coli* and *Pseudomonas*, human papilloma virus, and the pro-inflammatory bacteria *Propionibacterium acnes* [58]. However, as yet, no association between PCa and infection with any pathogen could firmly be established [59, 60]. Very recently, a link has been evoked between prostate cancer and the severe acute respiratory syndrome coronavirus 2 (SARS-CoV-2) infection, causing the current 2020 pandemic COVID-19. A gene named transmembrane serine protease 2 (TMPRSS2) for which alteration was historically associated with prostate tumorigenesis was recently confirmed to be involved in facilitating the SARS-CoV-2 entry into host cells. Yet, it is premature to speculate about any involvement of SARS-CoV-2 in PCa [61, 62].

In this study, we have searched for the presence of a panoply of urogenital mollicutes species (*M. hominis*, *M. genitalium, M. fermentans*, *U. urealyticum*, and *U. parvum*) in prostate tissues from KSA patients with PCa or BPH, using PCR coupled to nucleotide sequencing. In KSA, no studies have been undertaken so far dealing with the etiopathogenesis of PCa. The only available data are about the incidence and mortality rates related to this cancer [63]. Epidemiologic data relating to the time period 2001–2008 have reported a steady increase in PCa incidence and mortality rates [39], however, since then, the situation has not been updated. This further highlights the importance of PCa disease in KSA and prompts the investigation of a potential involvement of urogenital mollicutes in the progression of this cancer.

PCR results showed no amplification of *M. genitalium*, *M. fermentans*, or *M. hominis* DNAs from PCa tissues of Saudi patients. Such a result could be subject to variation, since in Iran the same study confirmed the absence of *M. genitalium* in PCa [30], while in Australia and Japan, *M. genitalium* was identified with rates of 8% [31] and 40% [29], respectively. *M. hominis* was detected at a rate of 15% in Russian men suffering from PCa [33], but not in Japanease patients [29]. Regarding *M. fermentans* species, it was suspected in the prostate carcinogenesis in Turkey, but no precise results about its prevalence were communicated [32].

A total of 15 Saudi patients tested were found to harbor *Ureaplasma* spp. DNA. All of them were diagnosed with PCa. Twelve patients were infected with only one *Urealplasma* species while the 3 others were co-infected with both *Ureaplasma* species simultaneously. *U. urealyticum* was detected in 8 among the 50 PCa patients (16%). This rate is the highest among the reported cases. Indeed, only one case of positive *U. urealyticum* PCR was reported in each of the Australian and the Iranian studies already mentioned above [30, 31]. In this study, *U. parvum* was identified at a rate of 20% (10/50). This is the first study that reports the detection of this species in patients with PCa. Furthermore, the coinfection with *U. urealyticum* and *U. parvum* of the same patient with PCa is a new finding reported for the first time by our study. However, this result remains to be consolidated. Apart from some new clues unveiled by our study, we found some similarities with other studies in terms of species identification and rates. This could explain the role played by several factors such as hereditary susceptibility, environmental factors, or even the methods used to identify *Mycoplasma* species and their genes [64]. The fact that we have detected *Ureaplasma* spp. only in patients suffering from PCa lends further support to the hypothesis suggesting their involvement in the etiology of this type of cancer.

In the case of our study, though the results are statistically supported, one can not formally conclude that the presence of *Ureaplasma* spp. is the primary cause of prostate carcinogenesis. However, according to the Gleason score values (8–10), the presence of *Ureaplasma* spp. could have contributed to the worsening of cancer progression. Indeed, patients with PCa harbouring ureaplasmas displayed invasive malignant tumours of the prostate. These findings are recorded in patients over 60 years old and with PSA values > 4 ng/ml. Like viruses, mycoplasmas are notoriously known for their ability to cause low-grade chronic inflammation and silent infection, a hallmark thought to gradually compromise the immune system of their hosts, thereby promoting cancer transformation. Nevertheless, their detection in tumour microenvironment does not imply that they are causally involved in carcinogenesis. Actually, many factors like the high prevalence of the infectious agent in the general population, the extended latency, the asymptomatic infections, and the immune system status of patients could make difficult establishing a causality link [9]. Also, one can not ignore the idea that detection of mycoplasma (or any other microorganism) in cancerous tissues could be a consequence of the compromised immune response in tested patients since cancer is known for its association with a wide range of immunological disorders. Indeed, it was suggested that viruses could be activated as consequence of carcinogenesis rather than being a cause of it, and that their carcinogenic potential could be related to the dramatically compromised immune system [9].

To sum up, the role of microbes in cancer is still a subject of debate and it is obvious that all these results further lead us to provide more evidence of the role that mollicutes can play in prostate cancer. Appropriate animal models and in vitro cell cultures could be very useful to achieve this goal.

## Conclusions

To our knowledge, this study is the first to be conducted in KSA aiming to characterize the PCa from an etiological standpoint. The finding that only patients with PCa were significantly associated with *Ureaplasma* spp. infection is in favor of their potential involvement in prostate cancer or, most likely, points to their contribution in worsening its progression. These data are worth to be considered by the Saudi health policy makers during decision-making processes regarding the control and follow-up of PCa epidemiology in KSA.

## Supplementary Information

**Additional file 1:** **Figure S1.** PCR amplification of *Ureaplasma parvum* 16S rRNA gene. Lane M: 1 kb + DNA ladder. Lanes 1–5: Specificity of primers of *U. parvum* 16S rRNA gene with DNA from human mitochondrion, *M. hominis*, *M. fermentans*, *M. genitalium*, and *U. urealyticum*. Lane 6: No DNA (negative control). Lane 7: DNA from *U. parvum* type strain ATCC 27845 (positive control). Lanes 8–15: DNA from prostate biopsies of patients (PCa cohort).

**Additional file 2:** **Figure S2.** PCR detection of *Ureaplasma urealyticum* 16S rRNA gene. Lane M: 1 kb + DNA ladder. Lanes 1–5: Specificity of primers of *U. urealyticum* 16S rRNA gene with DNA from human mitochondrion, *M. hominis*, *M. fermentans*, *M. genitalium*, and *U. parvum*. Lane 6: No DNA (negartive control). Lane 7: DNA from *U. urealyticum* type strain ATCC 27618 (positive control). Lanes 8–15: DNA from prostate biopsies of patients (PCa cohort).

## Data Availability

All data generated and analysed during this study are included in this published article and its supplementary information files. Materials are available upon reasonable request.

## Abbreviations

BPH: Benign Prostate Hyperplasia
DRE: Digital Rectal Examination
ExoSAP: Exonuclease Shrimp Alkaline Phosphatase
FFPE: Formalin-Fixed Paraffin-Embedded
KSA: Kingdom of Saudi Arabia
PCa: Prostate Cancer
PSA: Prostate Specific Antigen
TRUS: TransRectal UltaSound
TURP: TransUrethral Resection of the Prostate
WHO: World Health Organization
