# Supplementary figures and images for: Molecular detection of urogenital mollicutes in patients with invasive malignant prostate tumor
Source: Infect Agent Cancer. 2021 Jan 20;16:6. doi: 10.1186/s13027-021-00344-9 (PMC7816065; doi:10.1186/s13027-021-00344-9)

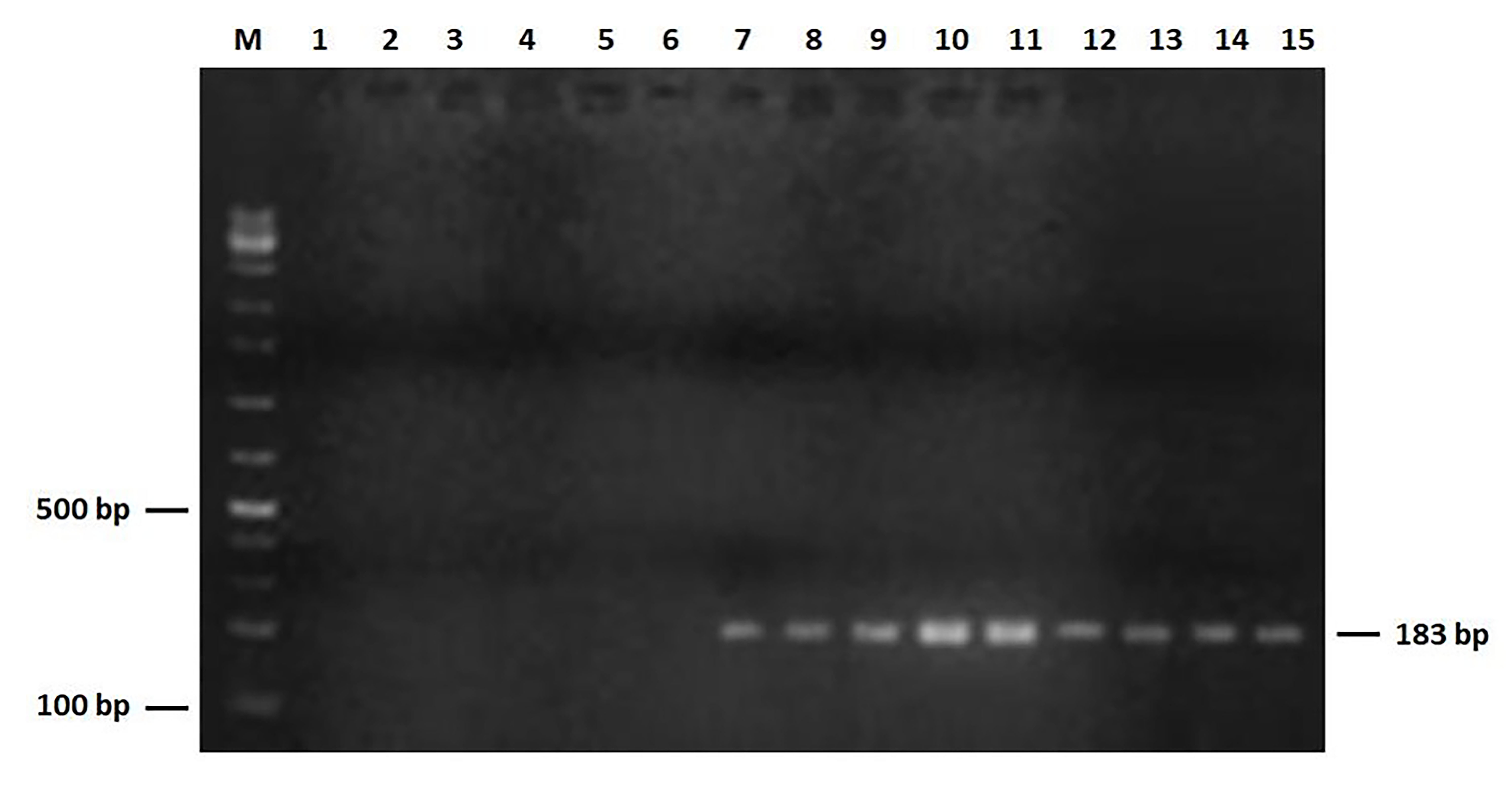

Supplement: Supplementary file 1 — Additional file 1: Figure S1. PCR amplification of Ureaplasma parvum 16S rRNA gene. Lane M: 1 kb + DNA ladder. Lanes 1–5: Specificity of primers of U. parvum 16S rRNA gene with DNA from human mitochondrion, M. hominis, M. fermentans, M. genitalium, and U. urealyticum. Lane 6: No DNA (negative control). Lane 7: DNA from U. parvum type strain ATCC 27845 (positive control). Lanes 8–15: DNA from prostate biopsies of patients (PCa cohort). [file 13027_2021_344_MOESM1_ESM.jpg]

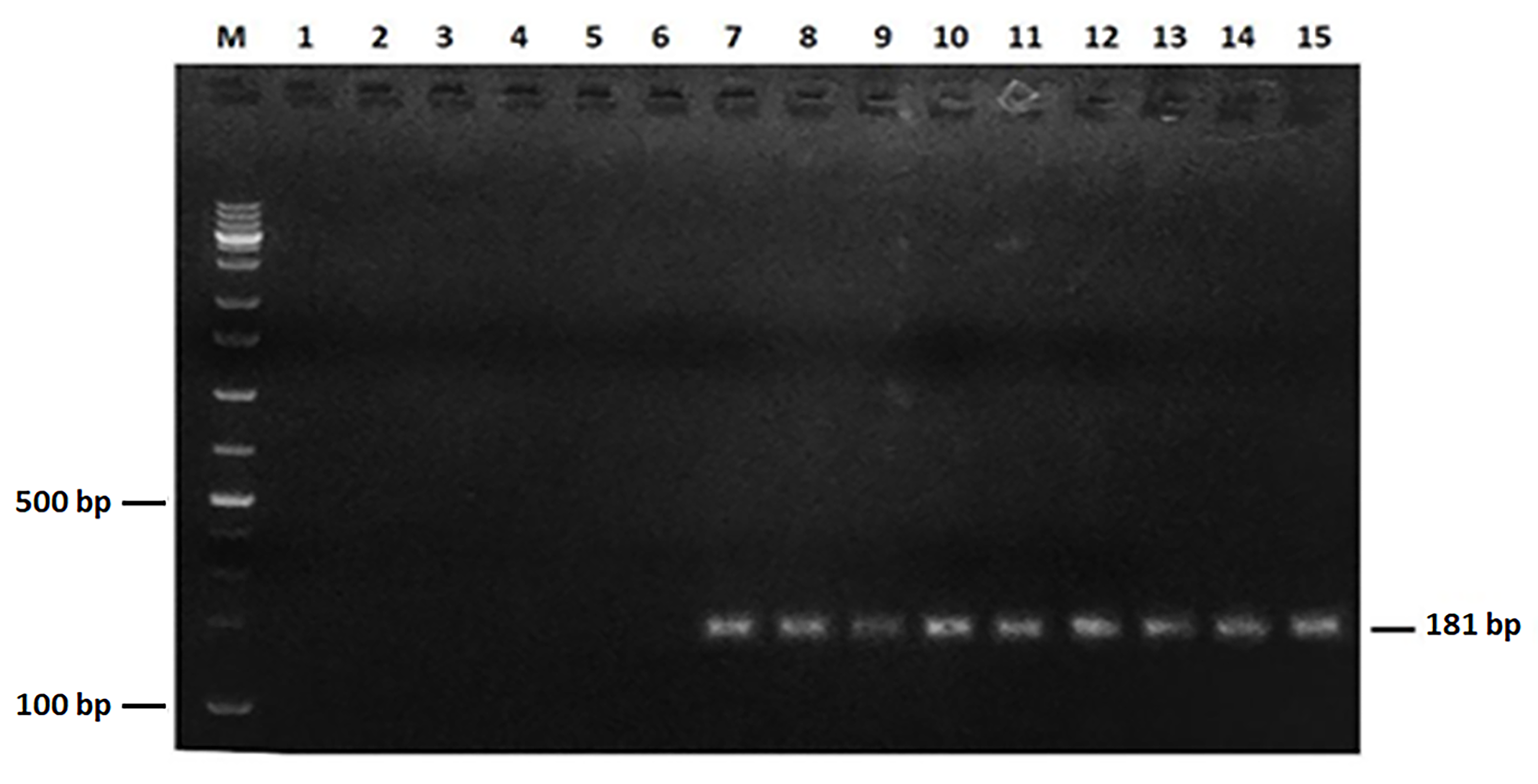

Supplement: Supplementary file 2 — Additional file 2: Figure S2. PCR detection of Ureaplasma urealyticum 16S rRNA gene. Lane M: 1 kb + DNA ladder. Lanes 1–5: Specificity of primers of U. urealyticum 16S rRNA gene with DNA from human mitochondrion, M. hominis, M. fermentans, M. genitalium, and U. parvum. Lane 6: No DNA (negartive control). Lane 7: DNA from U. urealyticum type strain ATCC 27618 (positive control). Lanes 8–15: DNA from prostate biopsies of patients (PCa cohort). [file 13027_2021_344_MOESM2_ESM.jpg]
